# Modelling the Combined Effects of Oxalic Acid, Water Activity, and pH on the Growth and Mycotoxin Production of *Aspergillus* spp. in a Dried Fig System

**DOI:** 10.3390/foods14223854

**Published:** 2025-11-11

**Authors:** Cristina Hidalgo, Alicia Rodríguez, Manuel J. Serradilla, Alberto Martín, Santiago Ruiz-Moyano

**Affiliations:** 1 Departamento de Producción Animal y Ciencia de los Alimentos, Nutrición y Bromatología, Escuela de Ingenierías Agrarias, Universidad de Extremadura, Avda. Adolfo Suárez s/n, 06007 Badajoz, Spain; cristinahr@unex.es (C.H.); amartin@unex.es (A.M.); srmsh@unex.es (S.R.-M.); 2 Instituto Universitario de Investigación de Recursos Agrarios (INURA), Universidad de Extremadura, Campus Universitario, Avda. de la Investigación s/n, 06006 Badajoz, Spain; 3 Área de Postcosecha, Valorización Vegetal y Nuevas Tecnologías, Centro de Investigaciones Científicas y Tecnológicas de Extremadura (CICYTEX), Instituto Tecnológico Agroalimentario de Extremadura (INTAEX), Junta de Extremadura, Avda. Adolfo Suárez s/n, 06007 Badajoz, Spain; manuel.serradilla@juntaex.es

**Keywords:** modelling, semi-quantitative predictive tool, mycotoxins, fungal growth, elicitor

## Abstract

This study aimed to model the effects of a_w_, pH, and OA, a compound commonly used as a plant elicitor, on the growth and mycotoxin production of *Aspergillus welwitschiae* and *Aspergillus flavus* on a fig-based model substrate. Using RSM with a BBD, the combined impact of a_w_ (0.92–0.99), pH (5.6–6.3), and OA (1–2 mM) on growth and mycotoxin production was evaluated under fixed temperature cycle simulating field conditions. HPLC-FLD quantified OTA and AFs. The results revealed that a_w_ was the most influential factor governing fungal behaviour. The driest a_w_ (0.92) significantly delayed growth and completely inhibited the production of OTA and AFB_1_. Conversely, high a_w_ (0.99) was a prerequisite for significant mycotoxin accumulation. While OA at the tested elicitor concentrations did not prove to be a potent independent inhibitor of mycotoxins, its interactions with aw and pH did significantly delay fungal growth. The high R^2^ values (>96%) for growth models indicated a strong goodness-of-fit for comparing the relative impact of the factors. The models for mycotoxins had more moderate R^2^ values, a common finding reflecting the complexity of secondary metabolism. Consequently, these models should be regarded as semi-quantitative tools for identifying high-risk trends rather than for precise prediction. Following internal validation, all developed models proved to be valuable semi-quantitative tools for identifying high-risk conditions, including those with more modest R^2^ values like the OTA model (R^2^ = 56.5%, validation R > 0.945).

## 1. Introduction

The fig tree (*Ficus carica* L.) is an important crop in the Mediterranean area [1] with an annual production exceeding 1 million tonnes. Turkey is the leading producer, followed by Egypt, Algeria, Morocco, Iran, and Spain [2]. Extremadura is Spain’s central fig-producing region, with a 2022 output of over 24,000 tonnes, primarily in the form of dried figs [3].

The traditional method for processing dried figs involves allowing the fruit to fully ripen and partially dehydrate both on the tree and, subsequently, on the ground. After harvest, the figs are typically sun-dried to a moisture content below 26%, following Dry and Dried Produce—Standards 14 [4], either in on-farm drying rooms, on cement floors, or using wire mesh systems [5]. However, this traditional processing method, combined with the high sugar content, makes figs more susceptible to fungi contamination, particularly during sun drying, which promotes mycotoxin accumulation [6].

Fungal infection in dried figs can occur both on the external surface of the fruit and internally, often facilitated by insect vectors under favourable temperature and humidity conditions during the drying process [7]. The most prevalent moulds associated with dried figs belong to the *Aspergillus* section *Nigri*, followed by *Fusarium* spp., *Aspergillus flavus*, *Penicillium* spp., *Alternaria* spp., and *Mucor* spp. [6,7,8,9,10]. In the Extremadura region, the main mycotoxigenic fungal species reported from field to fork in the dried fig processing are *A. welwitschiae* and *A. flavus*, known producers of OTA and AFs, respectively. Consequently, their mycotoxins have also been found from pre-harvest to storage stages [9,10,11,12]. Thus, mycotoxin contamination is a significant issue for commercial dried figs and their derivatives.

From January 2020 to December 2024, approximately 360 notifications concerning the presence of mycotoxins in dried figs have been reported at the Rapid Alert System for Food and Feed, with AFs being the most common, followed by OTA, mainly from Turkey and to a lesser extent from Spain [13]. In response to these risks, the European Commission has established maximum permissible limits for AFs in dried figs of 6 μg/kg for AFB_1_ and 10 μg/kg for total AFs (sum of AFB_1_, AFB_2_, AFG_1_, and AFG_2_) and 8 μg/kg for OTA [14].

Due to the development of toxigenic moulds and mycotoxin production in dried figs that begins in the field, various preharvest strategies have been implemented, including good agricultural practices outlined by the World Health Organisation [15]. The most effective method at this stage to manage the growth of filamentous fungi and subsequent mycotoxin contamination is the application of synthetic fungicides. However, their use is becoming increasingly restricted by stringent regulations due to concerns about environmental pollution, the development of resistance in toxigenic fungi and other significant plant pathogens, and adverse effects on human health [16,17,18]. Moreover, consumer demand for high-quality and safe products that are free of synthetic additives is increasing [19]. Consequently, recent research has focused on exploring safe and eco-friendly alternatives.

Among alternative preharvest strategies, the use of elicitors or biostimulants has recently been demonstrated to enhance the physicochemical and nutritional quality and the enzymatic and non-enzymatic antioxidant activity of fruits [20]; however, to the best of our knowledge, their potential effects on the hygienic-sanitary quality of fruits—particularly regarding the growth of toxigenic fungi—remain largely unexplored. One of the elicitors used is OA, a common organic acid found in plant tissues [21]. OA application, both preharvest and postharvest, has been found to enhance fruit quality at harvest and delay senescence during storage [21,22,23]. Some studies have shown that OA exhibits fungicidal activity and induces defence mechanisms against fungal pathogens in plants. For instance, the application of 3 mmol/L OA has been found to enhance tomato plants’ resistance to mechanical injury and infection by *Botrytis cinerea* [24]. Preharvest spraying of 5 mM OA on kiwifruit plants controls *Penicillium expansum* and patulin accumulation by increasing the activities of defence-related enzymes [25]. The OA dose needs to be adjusted depending on the genotype because it can also promote fungal infection or induce resistance [24]. However, a significant research gap exists regarding not only the direct impact of OA on toxigenic fungi relevant to figs, but critically, its interactive effects with key abiotic factors such as a_w_ and pH.

The evaluation of such interactions is crucial for developing effective control strategies. As shown in several studies, RSM has promise in this regard because it permits the manipulation of crucial parameters to maximise the study of the effect of the interaction of several compounds and abiotic factors [26,27]. BBD is an RSM, a mathematical and statistical tool for investigating and comprehending the complex connection between several variables and how they affect a specific response. Recent research supports the effectiveness of RSM in the development, enhancement, and optimisation of complex processes. This approach stands out for its economic efficiency because it provides a lot of data and information while drastically lowering the number of experiments required. Furthermore, as previous studies have demonstrated, RSM facilitates the identification of optimal conditions for achieving the desired response by enabling the analysis of the combined impact of various components and predicting the system’s response to new circumstances [28,29]. These models provide mathematical frameworks for predicting growth and mycotoxin production, and they also facilitate a comprehensive understanding of the complex interplay between the variables [30]. The use of mathematical models for quantifying and predicting behaviour may be helpful to ensure food safety [31]. Recently, numerous studies have focused on applying predictive growth to avoid fungal spoilage at early stages. Since the presence of mould in food is generally undesirable, the modelling of lag time before colony formation and even fungal growth rate could be of special interest in food safety management. Additionally, mycotoxin risk can be estimated through modelling [32]. Modelling the impact of temperature, a_w_, pH, and antifungals on *Penicillium* spp. and *A. flavus* and *A. carbonarius* growth and OTA and aflatoxin production has been studied on different food-based media [33,34,35,36,37]. Despite these advances, there is a notable lack of predictive models tailored specifically to the unique biochemical and physical characteristics of fig-based matrices. Furthermore, this absence of tailored models creates a disconnect between in vitro findings and their practical application, highlighting the need for mechanistic and practical insights that link predictive modelling to real-world contamination control in the fig industry.

In this context, this research aimed to (i) study the combined effects of OA, a_w_, and pH at temperatures commonly found in agricultural settings on the growth of *A. weltwitschiae* and *A. flavus* and their subsequent mycotoxin production. The optimisation of OA, a_w_, and pH parameters was carried out through RSM; (ii) develop and validate mathematical models to predict the growth and mycotoxin production of both toxigenic fungal species in conditions that match those for dried figs using RSM.

## 2. Materials and Methods

### 2.1. Fungal Strains and Inoculum Preparation

The ochratoxigenic *A. weltwitschiae* CVJ63 and aflatoxigenic *A. flavus M*144, both isolated from dried figs of the ‘Calabacita’ variety and obtained from the CAMIALI group at the University of Extremadura (Spain), were used in this study. *A. flavus* M144 has been previously identified and used by Galván et al. [38,39]. In contrast, *A. welwitschiae* CVJ63 has not been previously studied and was selected because it is one of the main fungal species found in dried figs in the Extremadura region [9,10]. Mould strains were routinely cultured on Potato Dextrose Agar (Condalab, Madrid, Spain) at pH 3.5 ± 0.1, adjusted with 1% (*v*/*v*) of tartaric solution at 10% (*w*/*v*). The plates were incubated at 25 °C for approximately 7 days until sporulation occurred. Spores of each strain were harvested by adding 10 mL of a sterile 0.05% (*v*/*v*) Tween 80 solution (Scharlab, Barcelona, Spain). The surface of each mould colony was gently swept, and the suspension formed was filtered through two layers of cheesecloth. The spore suspension was measured using a haemocytometer and adjusted to 5 × 10^5^ spores/mL with sterile distilled water for further use.

### 2.2. Experimental Design

A BBD with three factors and three blocks was applied to model the influence of the independent variables a_w_, OA concentration, and pH on the growth and mycotoxin production of *A. weltwitschiae* CVJ63 and *A. flavus M*144 (Table 1). The physicochemical (a_w_ and pH) parameter ranges were defined taking into account the drying process of figs [5] and the a_w_ range of dried figs that allows adequate mould growth for modelling, based on previous studies in vitro in *Aspergillus* spp. [40,41]. Regarding OA concentration, it was defined considering the most common preharvest concentrations of this elicitor applied at preharvest in other fruits [20].

### 2.3. Experimental Settings

A SSFB culture medium was used to conduct the assay. Using a Thermomix cooking robot, the pulp of ‘Calabacita’ fresh figs was combined with distilled water in a 1:1 (*w*/*v*) ratio, ground for five minutes at maximum speed, and then filtered using Whatman No. 1 filter paper to remove any solid residue. After one minute of boiling, the resultant fig juice was frozen at −80 °C until it was needed. The SSFB medium composition consisted of 25% (*v*/*v*) fig juice, 1% (*v*/*v*) of a 5% (*w*/*v*) yeast extract (Condalab) solution, and 3.2% (*v*/*v*) of a 0.4% (*w*/*v*) bacteriological agar (Condalab) solution. The a_w_ (from 0.99 to 0.92) of the SSFB medium was modified by substituting water with glycerol solution in water (45% *w*/*v*; Scharlab, Barcelona, Spain). The pH (from 6.3 to 5.6) was adjusted with a sodium hydroxide solution at 1 M (Scharlab). The different OA concentrations were obtained by spiking various amounts of OA solution at 0.2 M (Merck, Darmstadt, Germany). Every medium condition was inoculated at 1% with 5 × 10^5^ spores/mL suspension of the corresponding strain. As BBD was used to build the experimental model. The design consisted of 13 unique conditions: 12 factorial points and one central point. The central point was replicated three times to estimate the experimental error, resulting in a total of 15 runs per mould (Table 1). Non-inoculated controls for each condition were also prepared. The experiment was conducted as two independent biological replicates. Within each biological replicate, ten technical replicates were prepared for each treatment condition. To avoid potential edge effects, data from the two outermost wells were discarded. The final response for each experimental run was therefore the mean of the 8 central technical replicate wells.

Two hundred and fifty µL of each condition (inoculated media and non-inoculated controls) were pipetted into each well of the 100-well Bioscreen Honeycomb plates (Thermo Fisher Scientific, Waltham, MA, USA). The following temperature cycle was used to incubate the plates: after 2.5 h of a temperature cycle at 20 °C, the multiwell plates were incubated for 9.5 h at a temperature gradient of 0.2 °C every 10 min until 31.4 °C was reached. It was then maintained at 31.4 °C for 2.5 h before gradually dropping to 20 °C for 9.5 h at a rate of 0.2 °C every 10 min. For ten days, the 24-h temperature cycle was repeated. The temperature cycle was not included as a factorial variable in this study because our primary aim was to simulate a realistic environmental scenario in an agricultural context. This temperature range was selected to represent the average meteorological conditions recorded over the past five years during the fig harvest season in ‘Almoharín’, Spain [42]. The OD at 600 nm was recorded every 30 min without shaking. The equipment manufacturer’s “Easy Bioscreen Experiment” (EXEcperiment) software version 3.0.0.66 was used to collect the absorbance data, which were then exported to a Microsoft^®^ Excel 365 datasheet (Microsoft Corporation, Redmond, WA, USA) for further analysis.

#### 2.3.1. Growth Curve Analysis

##### Building Growth Curves

Before processing, the raw datasets from the Bioscreen C underwent two additional processes. First, the mean of the measurements from each well during the first 120 min was calculated and automatically subtracted from all subsequent measurements to eliminate different signal backgrounds. The OD readings at 600 nm were used to build the growth curves of *A. welwitschiae* CVJ63 and *A. flavus* M144 for each experimental condition using Microsoft^®^ Excel software.

##### Lag Phase, µ_max_, and the TTD at a Specific OD_600nm_ Value

The normalised Bioscreen C data as described in Section Building Growth Curves was used to obtain µ_max_ (OD_600nm/day_), and lag phase (hours) [43] using Microrisk Lab. v1.2. The Baranyi model, as the primary model, was applied to fit the Bioscreen C data according to Mytilinaios et al. [44].

The TTD for 0.25, 0.5, 1, 1.25, 1.5, 1.75, and 2 OD_600nm_ values was calculated using linear interpolation between consecutive OD measurements using a Microsoft^®^ Excel template kindly provided by Dr. R. Lambert [45].

#### 2.3.2. Mycotoxin Assessment

##### Extraction of Mycotoxins

After the 10-day incubation period, OTA and AFs were extracted. A methanol solution was used to extract OTA, while AFs were extracted with a solution of acetonitrile, water, and formic acid (79:20.9:0.1, *v*/*v*/*v*). All solvents used for extraction and quantification were HPLC grade (Thermo Fisher Scientific). The content of each well was placed in a 2 mL tube, weighed, and stored at −80 °C until extraction. Afterwards, 0.92 mL of solution extraction was added. Suspension was vortexed for 30 s and shaken for 90 min at 300 rpm at 25 °C in the dark. Subsequently, samples were centrifuged for 10 min at 13,000× *g*, filtered through a 0.22 µM pore size filter, and 600 μL of supernatant was transferred to amber glass HPLC vials for quantification. For the analysis of AFs, the 600 μL supernatant was diluted 1:1 with an acetonitrile/water/formic acid solution (20.9/79/0.1, *v*/*v*/*v*) before filtration to minimise potential matrix effects and improve chromatographic resolution and minimise potential matrix effects. This standardised protocol, which was fully validated for the fig-based matrix as described below, ensures consistent extraction efficiency and comparability across all experimental conditions.

The concentration of both mycotoxins was determined with an Agilent 1260 Series Infinity II HPLC instrument (Agilent Technologies, Santa Clara, CA, USA) coupled to an FLD (Agilent).

##### OTA Quantification

OTA was determined following the methodology described by Danial et al. [46]. Chromatographic separation was performed with a RESTEK RAPTOR C-18 column (15 cm × 4.6 mm, particle size 2.7 µm; Restek, Bellefonte, PA, USA) at 25 °C. The mobile phase contained a mixture of water: acetonitrile: acetic acid (41:57:2 *v*/*v*/*v*), which was delivered at an isocratic rate of 0.6 mL/min. The FLD detector excitation and emission wavelengths were 333 and 460 nm. OTA was eluted at 4.40 min. The signals were processed by Agilent Open Lab CDS Workstation Software version 3.2.0.620 (Agilent). OTA was quantified based on the HPLC fluorimetric response compared to a standard supplied by Thermo Fisher Scientific. The LOD of the analysis was 0.014 µg/kg, and the limit of quantification (LOQ) was 0.042 µg/kg.

##### AF Quantification

AF concentration was evaluated following the methodology described by Galván et al. [39]. Chromatographic separations were performed using a SUPELCOSIL LC-18 column (15 cm × 4.6 mm, 5-µm particle size; Supelco, Bellefonte, PA, USA) after post-column derivatisation with pyridinium bromide at 0.005% (*w*/*v*; Merck). The post-column derivatisation reagents were pumped at 0.3 mL/min using an HPLC pump from an Agilent 1260 Series Infinity II apparatus. The mobile phase contained a mixture of methanol: acetonitrile: water (20:20:60 *v*/*v*/*v*), which was delivered at an isocratic flow rate of 1 mL/min. AFB_2_ and AFB_1_ were eluted at 6.49 and 7.87 min, respectively. The FLD detector was set to excitation and emission wavelengths of 360 and 430 nm, respectively. The injection volume was 15 μL. The signals were processed by Agilent Open Lab CDS Workstation Software (Agilent), and calibrations were carried out with an AFs mix standard (AFB_1_, AFB_2_, AFG_1_, and AFG_2_) supplied by Merck. The LODs of the analysis were 0.010 µg/kg for AFB_1_ and 0.025 µg/kg for AFB_2_, based on a signal-to-noise ratio of 3:1. The LOQs were 0.03 µg/kg for AFB_1_ and 0.075 µg/kg for AFB_2_.

### 2.4. Data Analysis

#### 2.4.1. Statistical Analysis of Growth and Mycotoxin Data

Once the TTDs, lag phase, µ_max_ and mycotoxin data were obtained, ANOVA was performed using the different concentrations of OA (1–2 mM), pH (5.60–6.30) and a_w_ (0.92–0.99) as independent variables to evaluate the effect of OA, pH and a_w_ on the fungal growth and mycotoxin production of both toxigenic strains. The mean comparisons for each independent variable were done using Tukey’s HSD. For all analyses, the assumptions of normality and homoscedasticity were verified by examining the residual plots; no significant deviations were found. The StatGraphics Centurion XVI Version 16.1.18 software was used in the analysis.

#### 2.4.2. Creation of Mathematical Models

Mathematical modelling and optimisation were performed using StatGraphics Centurion XVI Version 16.1.18 software. BBD with three factors, 3 blocks, and 15 experimental runs (12 as factorial points and 3 as middle points; Table 1). The experimental data designed by BBD were analysed by the response surface regression procedure. The quadratic model (Equation (1)) was as follows:

(1) $$Y=\beta_{0}+\beta_{1}X_{1}+\beta_{2}X_{2}+\beta_{3}X_{3}+\beta_{12}X_{1}X_{2}+\beta_{13}X_{1}X_{3}+\beta_{23}X_{2}X_{3}+\beta_{11}X_{1}^{2}+\beta_{22}X_{2}^{2}+\beta_{33}X_{3}^{2}+\varepsilon$$

where *Y* is the response variable predicted by the model; *β*_0_ is a constant value; *β*_1_, *β*_2_ and *β*_3_ are the regression coefficients for the main (linear) terms; *β*_11_, *β*_22_, and *β*_33_ are quadratic effects; *β*_12_, *β*_13_, and *β*_23_ are interaction effects; *X*_1_ and *X*_2_ are independent variables, and ε is the experimental error.

An ANOVA was performed to evaluate the independent effects of the three factors (AO, a_w_, and pH), their quadratic effects, and the interactions between the factors, establishing statistical significance at a 95% confidence level.

##### Model Validation

To validate the mathematical models obtained, *A. weltwitschiae* CVJ63 and *A. flavus* M144 were inoculated onto the SSFB culture medium adjusted to different 5 combinations of a_w_ × AO × pH (Table 2), and the experimental settings were as described in Section 2.3. The a_w_ values and AO concentrations were intermediate between those used for the construction of the mathematical model. The pH was kept constant, since no significant effect on either growth or mycotoxin production was observed when the mathematical modelling was constructed. The lag phase, µ_max_, and TTD calculations were carried out as detailed in Section 2.3.1. Mycotoxins were determined at the end of the incubation period as described in Section 2.3.2.

The models were validated using an independent dataset of five experimental runs (Table 2) to test their interpolative power. The specific factor combinations were chosen to be within the original experimental domain but were not part of the initial BBD design. As our models indicated that pH was the least influential factor, it was kept relatively constant, while a_w_ and OA were varied using new combinations. Regarding the process, the validation experiments were conducted first to generate observed data. The model’s predictions for these specific factor combinations were then generated and compared against the experimental outcomes. Experimental and predicted values were correlated using a simple regression using StatGraphics Centurion XVI Version 16.1.18 software.

## 3. Results

### 3.1. Growth

#### 3.1.1. Growth Curves for *A. welwitschiae* and *A. flavus*

**Figure 1:**
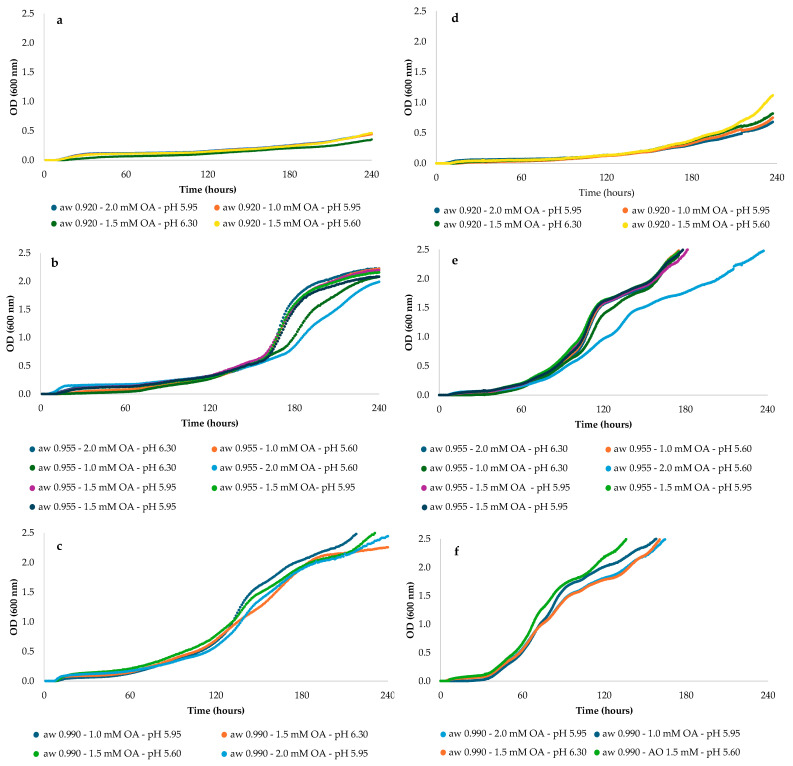
Mean growth curves at OD_600nm_ of *A. welwitschiae* (**a**–**c**) and *A. flavus* (**d**–**f**) at 0.92 a_w_ (**a**,**d**), 0.95 a_w_ (**b**,**e**), and 0.99 a_w_ (**c**,**f**) for 15 tested conditions.

Figure 1 shows the growth curves at 600 nm over time of *A. welwitschiae* and *A. flavus* for the 15 tested conditions (Table 1; Figure S1). The impact of conditions on mycotoxigenic fungi varies, with *A. flavus* strain showing greater sensitivity than *A. welwitschiae*. The growth curves of each mould species could be grouped into three categories, mainly based on a_w_ tested, due to the differences shown in their shapes. The shortest lag phase was observed at the wettest a_w_ (0.99), while the most extended lag phase was observed at the driest a_w_ (0.92). In the case of the lowest a_w_ evaluated, practically no growth was observed in any of the toxigenic moulds, regardless of the species, OA concentration, and pH value (Figure 1).

#### 3.1.2. Effect of Conditions on Lag Phase and µ_max_

Table 3 shows the *p*-values of the ANOVA applied to evaluate the effects of the factors a_w,_ OA concentration, and pH level, and their interactions, as well as the correlation of the model for lag phase and µ_max_ of *A. welwitschiae* and *A. flavus*. The adjusted R^2^ values were quite good (>91.05%), demonstrating that the models explained most of the observed data variability. This result was consistent despite the inclusion of both significant and non-significant factors in the models.

Regarding lag phase, values ranged from 39.3 to 131.1 h for *A. welwitschiae* and from 20.2 to 179.1 h for *A. flavus* (Table S1). Table 3 displays that the a_w_ factor showed significant linear (negative) and quadratic (positive) effects for the lag phases of *A. welwitschiae* and *A. flavus*, estimating maximisation of the lag phase to 129.4 h under the lowest a_w_ (0.92) for *A. welwitschiae*. On the contrary, OA and pH, and other quadratic effects were not statistically significant (*p* > 0.05). There was a significant (negative) interaction between a_w_ and OA for the lag phase of *A. flavus*. This means that the lag phase of *A. flavus* was delayed to 178.2 h under conditions that included the maximum OA concentration (2 mM) and minimum a_w_ (0.92) studied.

Concerning maximum µ_max_, mean values ranged from 2.04 to 4.58 OD_600nm/day_ for *A. welwitschiae* and from 0.53 to 1.47 OD_600nm/day_ for *A. flavus* (Table S1). Table 3 shows that the a_w_ factor had a significant linear (positive) effect on µ_max_ of *A. flavus* and a quadratic (negative) effect in the case of both toxigenic strains. The minimum µ_max_ values for *A. flavus* were observed at 0.92 a_w_. In contrast, for *A. welwitschiae,* the µ_max_ values were observed at either the minimum or maximum a_w_ tested due to the quadratic effect of this factor. OA, pH, other quadratic effects, or interactions were not statistically significant (*p* > 0.05). There was a significant (positive) interaction between OA and pH in the models for µ_max_ of *A. welwitschiae*. This means that µ_max_ of *A. welwitschiae* was minimised to 1.37 OD_600nm/day_ under conditions of maximum OA concentration (2 mM), minimum pH (5.60), and maximum a_w_ (0.99). Conversely, no interactions were observed between the studied factors for *A. flavus*.

#### 3.1.3. Effect of Conditions on TTD of *A. welwitschiae* and *A. flavus*

The TTD is described as the necessary time for fungal growth to reach a specific OD_600nm_ level under each condition tested. Lower TTD values indicate faster growth, while higher values correspond to slower growth. Table 4 and Table 5 show the *p*-values of the ANOVA applied to evaluate the effects of the factors a_w_, OA concentration, and pH level, and their interactions, as well as the correlation of the model for TTD at OD_600nm_ values of 0.25, 0.5, 0.75, 1, 1.25, 1.75 and 2 of *A. welwitschiae* (Table 4) and *A. flavus* (Table 5). The adjusted R^2^ values were notably high (>93.30%) for all TTD calculated for OD_600nm_ from 0.25 to 2, indicating that the models explained most of the observed data variability.

The mean TTD values varied depending on the species, experimental conditions, and the OD_600nm_ value (Tables S2 and S3). For example, in the case of *A. welwitschiae,* average TTD values at OD_600nm_ of 0.25 ranged between 2219.0 and 6290.7 min (Table S2), while for *A. flavus,* they ranged between 2650.6 and 9901.9 min (Table S3). Among the factors analysed, both the linear (negative) and quadratic (positive) effects of a_w_ were the most influential in the model for TTD at the eight OD_600nm_ values evaluated for *A. welwitschiae* and *A. flavus* (Table 4 and Table 5). The model estimated the slowest mould growth (highest TTD value) at the lowest a_w_ evaluated (0.92 a_w_), while the estimated values for TTD minimisation were at high a_w_ levels (a_w_ > 0.98). The OA factor showed a significant linear (negative) effect for the TTD at OD_600nm_ of 0.25 just for *A. welwitschiae* (Table 4). In contrast, this factor had a significant quadratic (positive) effect for the TTD at OD_600nm_ from 0.25 to 0.75 for *A. flavus* (Table 5).

The model showed higher TTD values at the lowest OA concentration studied (1 mM) for *A. welwitschiae* and at the highest OA concentration (2 mM) for *A. flavus*, while an intermediate OA concentration (1.58–1.47 mM, respectively) was predicted for TTD minimisation (faster growth). The pH value showed no significant linear or quadratic effect. Regarding the interactions between factors, a_w_ showed no interaction with the other factors for both mould species tested.

On the other hand, there was a significant (negative) interaction between OA and pH in the models from TTD values at OD_600nm_ values from 1 to 2 in the case of *A. welwitschiae* and at 1.75 for *A. flavus*. This indicates that under conditions of the highest OA concentration tested (2 mM) and lowest pH specified (5.6), the growth of both strains was slowed at the end of the exponential phase (greater TTD value).

### 3.2. Effect of Conditions on OTA and AFs Accumulation

Table 6 shows the *p*-values of the ANOVA applied to evaluate the effects of the parameters a_w_, OA concentration, and pH level and their interactions, as well as the correlation of the model for OTA and AFs (AFB_1_ and AFB_2_) accumulation by *A. welwitschiae* and *A. flavus*, respectively, after 10 days. The adjusted R^2^ for OTA and AFs varied between 51.46 and 62.00%. The lack-of-fit test results were not significant for all mycotoxin models (*p* > 0.11).

Mean values for OTA ranged from 50.6 to 7856 µg/kg for *A. welwitschiae,* while those for AFB_1_ and AFB_2_ varied between 519.1 and 25,253.4 µg/kg and 27.0 to 616.2 µg/kg, respectively, for *A. flavus* (Table S1). The a_w_ factor showed a linear effect on all mycotoxins; however, in the case of OTA, the effect was negative, whereas for AFs, it was positive. In addition, a_w_ exhibited a quadratic (negative) impact in the case of AFB_2_. In contrast, no significant effects of OA, pH and their interactions on mycotoxin accumulation were observed. At the highest a_w_ evaluated (0.99), the estimated OTA accumulation was >6500 µg/kg, and AFB_1_ accumulation was >25,500 µg/kg. Nevertheless, at the driest a_w_ (0.92), OTA and AFB_1_ would not be produced either by the *A. welwitschiae* or *A. flavus* strain, respectively, although *A. flavus* would remain at minimal production of AFB_2_.

### 3.3. Predictive Models

Table 7 shows the polynomial equations obtained by applying RSM to predict growth parameters (lag phase, µ_max_, and TTD) and mycotoxins (OTA, AFB_1_, and AFB_2_) for *A. welwitschiae* and *A. flavus*, in which only the significant effects were considered as independent variables. The correlation coefficient (R^2^) values for the prediction equations are also shown in Table 7. Regarding *A. welwitschiae*, the R^2^ values were higher than 97.9% for all the parameters analysed, except for µ_max_ and OTA, whose R^2^ values were 88.5 and 56.5%, respectively. In the case of *A. flavus*, the R^2^ values for TTDs and lag phase were higher than 96.6%, while for µ_max_, AFB_1_ and AFB_2_ were 90.3%, 63.4%, and 74.7%, respectively. The high R^2^ values of the equations in most cases indicate their strong predictive capability for the evaluated parameters under the studied factor values. It should be noted that in the case of TTD, those OD_600nm_ values with the most significance was selected for subsequent data validation, being TTD for 0.25, 0.50, and 1 OD_600nm_ for *A. welwitschiae* and TTD for 0.25, 1, and 1.75 OD_600nm_ for *A. flavus*_._

### 3.4. Validation of the Predictive Models

The developed models were validated with independent experimental data from those used for constructing the mathematical models (Table 2). *A. welwitschiae* and *A. flavus* were inoculated onto SSFB and incubated under conditions simulating dried fig processing at the preharvest stage. To corroborate the accuracy of the prediction equations obtained to estimate growth and mycotoxin production by the two toxigenic species, R values were calculated from experimental results and those predicted by the prediction equations (Tables S4–S6). Regarding *A. welwitschiae*, the R values were higher than 0.945 for all the parameters analysed, except for µmax, whose value was 0.524. In the case of *A. flavus*, the R values for TTDs and lag phase were higher than 0.960, while for µ_max_, AFB_1_, and AFB_2_ were 0.738, 0.584, and 0.242, respectively.

## 4. Discussion

The contamination of dried figs by mycotoxigenic fungi, particularly *Aspergillus* species, represents a significant challenge for food safety and international trade. This study aimed to model the combined effects of a_w_, OA concentration, and pH on the growth and mycotoxin production of ochratoxigenic *A. weltwitschiae* and aflatoxigenic *A. flavus* on a fig-based substrate.

One helpful technique for examining the effects of abiotic factors on mould behaviour in various experimental settings is RSM. It enables the systematic assessment of key factors that significantly impact fungal growth and mycotoxin formation, such as pH, temperature, a_w,_ or antifungal compounds. RSM makes it possible to manipulate and interact with various variables, which allows for a thorough evaluation of how these variables, together with the elicitor, impact fungal development. To our knowledge, this is the first time that this methodology has been used to evaluate the effect of such abiotic factors together with an elicitor on fungal development in a dried fig model system. So far, RSM has been utilised to optimise the conditions of different strategies to counteract fungi and mycotoxin production [47,48,49].

Temperature, a_w_, and pH are important variables that influence the growth of mould and the consequent formation of mycotoxin [50,51]. Although it should emphasise the important role of temperature-a_w_ interactions in predictive mycology, as these interactions strongly affect microbial growth [26], temperature was not an independent variable in our study. We established a temperature cycle that mimicked the summer day and nighttime temperatures that are typical during this fruit’s production in the Mediterranean area to simulate realistic field conditions. Therefore, the influence of the other two parameters was considered together with the impact of an elicitor, OA.

The fungal growth parameters were estimated from OD data (TTD, µ_max_, and lag phase), which is an indirect measure of biomass for filamentous fungi and does not directly reflect colony formation [52]. This method can be influenced by spore germination, hyphal density, and pigmentation, potentially affecting the biological interpretation of the lag phase and maximum growth rate [52]. Therefore, in this study, lag phase and µ_max_ values derived from OD were used as comparative indicators of treatment effects rather than as absolute biological measures. Indeed, these two kinetic parameters showed consistency with TTD results across the studied factors (a_w_, pH, OA).

a_w_ is a critical factor governing microbial growth, and this study confirms its significant influence on both *A. welwitschiae* and *A. flavus*. The delay in fungal growth observed at lower a_w_ levels is a well-documented phenomenon attributed to osmotic stress, which limits the water bioavailability essential for fungal metabolism and enzymatic functions. To the best of our knowledge, this is the first study to evaluate the growth of *A. welwitschiae* in a fig-based matrix. This is significant because *A. welwitschiae* is a relatively novel species that was previously misidentified as *A. niger* [53,54,55]. Consequently, there is limited research available for direct comparison. Our findings indicate that its behaviour is consistent with previous reports on other substrates. For instance, it has been shown that *A. welwitschiae* and *A. niger* exhibit similar in vitro growth patterns, with optimal growth at high a_w_ (0.99) and a significant delay in both lag and lag phases at lower a_w_ levels (e.g., 0.90). This highlights the high-water requirement for this species to thrive [56]. Similarly, for *A. flavus*, a_w_ is a key determinant of its growth and metabolic activity [57]. At lower a_w_ levels (e.g., 0.93), the fungus prioritises essential biological processes for survival [58]. In contrast, at higher a_w_ (e.g., 0.99), it can trigger additional metabolic pathways, including the production of AFs [59]. Previous research has demonstrated that low a_w_ (0.90) can delay the growth phases of *A. flavus* by as much as 50 to 100 h compared to higher a_w_ levels (0.945) at 25 °C [60]. Although both species exhibit sensitivity to water stress, direct comparison studies on their specific a_w_ thresholds within a single matrix are scarce, a deficiency that the current work aims to rectify.

Beyond a_w_, this study revealed that OA, both independently and in conjunction with a_w_ and pH, exerts a significant inhibitory effect on the growth of both mould species. However, its impact is less noticeable than that of a_w_. This is consistent with the known mechanisms of organic acids, whose efficacy is intrinsically linked to pH. The antimicrobial activity of weak acids is primarily attributed to the undissociated form, which can passively diffuse across the cell membrane and dissociate in the higher pH of the cytoplasm, leading to acidification and metabolic disruption. Acidophilic fungi, in turn, can enhance their pathogenicity by secreting organic acids, such as OA, which lowers the pH of the host tissue and can cause damage [61]. Interestingly, the optimal pH for the growth of *A. niger* and *A. flavus* has also been found to be ideal for OA production [62].

OA, which is a major secondary metabolite of many fungi, including *Aspergillus* species, has a dual role [63]. It can act as a virulence factor [64] or it can act as a growth inhibitor at specific concentrations. So far, the application of OA has shown promise in controlling fungal growth in various agricultural studies. Sun et al. [24] found that elevated levels of OA (20 mM) were correlated with increased invasion by *Botrytis cinerea* in tomato plants, while lower concentrations (3 mM) induced resistance. Another study demonstrated that preharvest spraying of kiwifruit plants with 5 mM OA has been shown to improve postharvest quality and inhibit the growth of *Penicillium expansum* and the accumulation of its mycotoxin, patulin [25]. So, our findings demonstrate that the same acid can become self-limiting or inhibitory.

Mycotoxin contamination of food commodities, such as dried figs, represents a significant food safety concern. OTA and AFs are notably the most frequently identified mycotoxins in ‘Calabacita’ dried figs. In a study carried out by Galván et al. [39] in a dried fig agar-based medium, *A. niger* and *A. flavus* M144 were observed to produce mean concentrations of OTA and total AFs (AFB_1_ + AFB_2_) of 8.57 ± 0.52 µg/kg and 2.87 ± 2.14 µg/kg, respectively, by day 8. Furthermore, after a 12-day incubation period in a dried fig-based medium, *A. flavus* M144 produced AFB_1_ in concentrations ranging from <LOD to 60.63 ± 7.70 µg/kg, and AFB_2_ from <LOD to 0.02 ± 0.01 µg/kg, across a temperature range from 16 to 37 °C.

In the present investigation, the a_w_ emerged as the sole environmental factor that significantly influenced mycotoxin production by both *A. welwitschiae* and *A. flavus*. Higher a_w_ (0.99) was a prerequisite for the synthesis of OTA by *A. welwitschiae*, whereas production was inhibited entirely at the lower a_w_ of 0.92. This corroborates the work of Abarca et al. [56], who also observed no OTA production by *A. welwitschiae* and *A. niger* at a_w_ 0.90. This strong dependence on high water availability was also evident for AFs production by *A. flavus*. Our results are in full agreement with the literature, which consistently demonstrates that while fungal growth may occur at moderate a_w_ levels, mycotoxin production requires more permissive conditions. For example, studies have shown that AFs production can be undetectable at a_w_ values around 0.92–0.94, yet reach substantial levels (e.g., >2000 µg/kg) when a_w_ is elevated to 0.98, particularly when combined with optimal temperatures (30–35 °C) [65,66]. This suggests that for mycotoxin synthesis, a_w_ acts as a critical switch, more so than a gradual modulator.

Interestingly, neither pH nor the tested concentrations of OA demonstrated a significant direct effect on OTA or AFs production in our model system. This finding is particularly significant given that previous studies have reported contrasting results. In this sense, Zhu et al. [25] found that preharvest application of OA inhibited patulin production by *P. expansum*, and Alcano et al. [67] observed that pH modulated OTA production by *A. niger*. The absence of such an effect in our study implies that either the concentration needed to inhibit toxin synthesis differs from those affecting growth, or that the impact of these factors on mycotoxin production is highly context-dependent and may be overshadowed by the dominant effect of a_w_. The fact that sub-lethal stress caused by chemical agents can occasionally increase the formation of mycotoxin is also important to take into account; although not observed in this study, it is crucial for risk assessment.

The development of robust and reliable predictive models is a key component of food safety management, providing essential tools to anticipate and mitigate risks associated with mycotoxigenic fungi [68]. In this study, RSM was employed to model the influence of a_w_, OA concentration, and pH on the growth and mycotoxin production of *A. welwitschiae* and *A. flavus*. The generated polynomial equations showed high R^2^, indicating a strong correlation between the independent variables and the predicted outcomes for fungal growth parameters and mycotoxin production. Specifically, for *A. welwitschiae*, R^2^ values exceeded 97.9% for most of the parameters, and for *A. flavus*, they were above 96.6% for TTD and lag phase. As is common in mycology modelling, the R^2^ values of the mycotoxin models were moderate, ranging from 56.5% to 74.7%. This disparity arises because fungal growth is a primary metabolic process that is relatively stable and predictable. In contrast, mycotoxin synthesis is a secondary metabolic pathway, which is inherently more variable and sensitive to subtle environmental signals, substrate composition, and gene expression that may not be fully captured by the main factors of a_w_, pH, and OA alone [69,70]. Importantly, despite the moderate R^2^ values, the lack-of-fit test for all mycotoxin models was not significant (*p* > 0.11 in all cases), providing strong statistical evidence that the second-order polynomial model was adequate for describing the response surface. Furthermore, the models successfully captured the key trends in toxin production, such as the risk being highest at optimal a_w_ and negligible at the inhibitory a_w_ of 0.92. Therefore, these models serve as valuable semi-quantitative tools for identifying high-risk scenarios. Given their moderate R^2^ values, they are intended for trend identification within the experimental scope, not for precise extrapolation beyond the modelled range.

The validation of these predictive models against independent experimental data is a critical step to ensure their practical applicability. In the present study, the high values observed between predicted and experimental data for most parameters of both *A. welwitschiae* (R > 0.945 for most parameters) and *A. flavus* (R > 0.960 for TTDs and lag phase) substantiate the reliability of the developed models. While the models for µ_max_ and mycotoxin production, particularly for *A. flavus*, showed weaker correlations, they still provide valuable insights. It should be noted that for OTA production by *A. welwitschiae*, despite a more modest R^2^ for the prediction equation (56.5%), the model demonstrated excellent predictive power during validation (R > 0.945). Our results align with some studies in the field of predictive mycology that have evaluated the suitability of models by plotting predicted versus observed values for various *Aspergillus* spp. For instance, Aldars-García et al. [65] found that prediction accuracy for *A. flavus* TTD was temperature-dependent, sometimes resulting in under- or overestimations. Similarly, Norlia et al. [66] reported that their models predicted the maximum µ_max_ for *A. flavus* acceptably, although the strains grew more slowly than predicted. The validation of the lag phase has also been the subject of attention by Garcia et al. [71], who validated models for *A. ochraceus* and *A. parasiticus* on food matrices such as maize, coffee, and peanuts.

Finally, the scope of our experimental design warrants consideration for the practical application of these models. Temperature was intentionally held constant under a controlled cycle to isolate the effects of a_w_, pH, and OA, which precludes the analysis of its interaction with other variables under real-world thermal fluctuations. Furthermore, this study was conducted on a fig-based model substrate to establish a foundational understanding of these complex interactions. Consequently, validation on real dried figs was outside the scope of the current study and remains a necessary next step before these models can be confidently applied in the field. This future work will be crucial for bridging the gap between our controlled findings and their implementation as a robust risk management tool.

Predictive mycology has become an increasingly important field, with numerous studies focusing on modelling the growth and mycotoxin production of various fungal species in different food matrices [72,73,74,75]. The models developed in this study contribute to the growing body of knowledge on mycotoxin management and provide a semi-quantitative, practical tool for risk assessment in the dried fig industry. By shedding light on how key environmental factors, together with antifungal compounds, affect fungal behaviour, these models help support safer food production and more informed decision-making.

## 5. Conclusions

From a food safety and mycology perspective, this study concludes that while the preharvest treatment with OA shows potential as part of a multi-hurdle approach, a_w_ remains the single most critical factor controlling the growth of *A. welwitschiae* and *A. flavus* and their respective mycotoxin production in a fig-based substrate. The experiments, conducted under fluctuating temperatures simulating field conditions, unequivocally demonstrated that maintaining a_w_ at or below 0.92 is an effective strategy to prevent not only fungal proliferation but, more importantly, the synthesis of OTA and AFB_1_. The application of OA at the tested concentrations of 1–2 mM did not prove to be a potent independent inhibitor of mycotoxin production. However, its utility lies in its significant interactive effects on fungal growth. Specifically, OA synergistically delayed the lag phase of *A. flavus* when combined with water stress and inhibited the µ_max_ of *A. welwitschiae* when combined with a lower pH (5.6). The high accuracy (R^2^ > 96%) of the developed models for growth parameters (lag phase, TTD) indicates a strong goodness-of-fit, supporting their use for comparing the relative impact of the factors on mould development. The models for mycotoxin production were, as expected, less precise than those for growth; however, it is particularly noteworthy that for OTA production by *A. welwitschiae*, despite a more modest R^2^ for the prediction equation (56.5%), the model performed well during internal validation (R > 0.945). This strong correlation supports its utility for interpolating risk within the tested range of conditions. These models can therefore assist the dried fig sector in producing safer food by serving as valuable semi-quantitative decision-support tools.

## Figures and Tables

**Table 1:** Experimental runs of the BBD.

a_w_	OA (mM)	pH
Factorials points		
0.990	1.5	6.30
0.955	2.0	5.60
0.990	2.0	5.95
0.990	1.0	5.95
0.920	2.0	5.95
0.920	1.0	5.95
0.955	2.0	6.30
0.955	1.0	5.60
0.920	1.5	6.30
0.955	1.0	6.30
0.990	1.5	5.60
0.920	1.5	5.60
Central points		
0.955	1.5	5.95
0.955	1.5	5.95
0.955	1.5	5.95

**Table 2:** Experimental runs for model validation.

a_w_	OA (mM)	pH
0.940	1.25	6.00
0.970	1.25	6.00
0.940	1.75	6.00
0.970	1.75	6.00
0.950	1.5	6.15

**Table 3:** ANOVA results for RSM and optimal conditions of lag phase (hours) and µ_max_ (OD_600nm/day_) of *A. welwitschiae* and *A. flavus*.

Factors	*A. welwitschiae*		*A. flavus*	
	Lag Phase	µ_max_	Lag Phase	µ_max_
*p*-values ^a^				
a_w_	0.000	0.181	0.000	0.000
OA	0.156	0.415	0.131	0.143
pH	0.219	0.106	0.834	0.566
a_w_ * a_w_	0.000	0.000	0.000	0.001
a_w_ * OA	0.480	0.628	0.037	0.631
a_w_ * pH	0.788	0.544	0.363	0.134
OA * OA	0.741	0.350	0.713	0.472
OA * pH	0.973	0.001	0.383	0.212
pH * pH	0.840	0.131	0.204	0.490
R^2^	99.73	96.84	99.63	96.80
Adjusted R^2^	99.25	91.15	98.98	91.05
Optimisation (MIN) ^b^				
a_w_	0.990	0.990	0.990	0.920
OA (mM)	1.00	2.00	2.00	2.00
pH	5.60	5.60	6.19	5.60
Optimal value	40.0 ^c^	1.37 ^d^	16.0 ^c^	0.40 ^d^
Optimisation (MAX) ^b^				
a_w_	0.920	0.955	0.920	0.977
OA (mM)	2.00	2.00	2.00	1.00
pH	6.30	6.30	5.60	5.60
Optimal value	129.4 ^c^	4.39 ^d^	178.2 ^c^	1.54 ^d^

^a^ *p*-values lower than 0.05 are statistically significant. Green values indicate positive effects, and red values indicate negative effects. ^b^ Optimisation means the combination of significant factors necessary to obtain the highest value (max) or the lowest value (min) of lag phase and µmax. ^c^ hours. ^d^ OD_600nm/day_. * Evaluation of quadratic effects of the factor (a_w_ * a_w_, OA * OA and pH * pH) and interaction effects between factors (a_w_ * OA, a_w_ * pH and OA * pH).

**Table 4:** ANOVA results for the RSM and optimal conditions of TTD at OD_600nm_ (0.25, 0.5, 0.75, 1, 1.25, 1.5, 1.75, and 2) of *A. welwitschiae*.

Factors	TTD							
	0.25	0.5	0.75	1	1.25	1.5	1.75	2
*p*-values ^a^								
a_w_	0.000	0.000	0.000	0.000	0.000	0.000	0.000	0.000
OA	0.025	0.826	0.499	0.382	0.353	0.241	0.257	0.558
pH	0.087	0.218	0.484	0.535	0.547	0.726	0.816	0.401
a_w_ * a_w_	0.000	0.000	0.000	0.000	0.000	0.000	0.000	0.000
a_w_ * OA	0.111	0.278	0.679	0.633	0.619	0.465	0.523	0.506
a_w_ * pH	0.224	0.352	0.271	0.251	0.791	0.881	0.381	0.076
OA * OA	0.341	0.345	0.347	0.201	0.359	0.357	0.254	0.961
OA * pH	0.842	0.922	0.169	0.026	0.026	0.014	0.004	0.043
pH * pH	0.372	0.841	0.587	0.228	0.167	0.112	0.056	0.307
R^2^	99.39	99.59	99.74	99.77	99.69	99.67	99.72	99.33
Adjusted R^2^	98.28	98.85	99.28	99.36	99.13	99.09	99.22	98.12
Optimisation (MIN) ^b^								
a_w_	0.990	0.990	0.990	0.987	0.985	0.983	0.981	0.979
OA (mM)	1.58	1.21	1.07	1.00	1.00	1.00	1.00	1.00
pH	5.92	5.60	5.76	5.66	5.60	5.61	5.65	5.68
Optimal value ^c^	2103	3071	3584	3826	3942	4138	4535	4940
Optimisation (MAX) ^b^								
a_w_	0.920	0.920	0.920	0.920	0.920	0.920	0.920	0.920
OA (mM)	1.00	1.00	1.00	1.00	1.00	1.00	1.00	1.01
pH	6.30	6.30	6.30	6.26	6.26	6.30	6.30	6.30
Optimal value ^c^	6563	8062	8904	9318	9587	9935	10,510	12,663

^a^ *p*-values lower than 0.05 are statistically significant. Green values indicate positive effects, and red values indicate negative effects. ^b^ Optimisation means the combination of significant factors necessary to obtain the highest value (max) or the lowest value of TTD (min). ^c^ minutes. * Evaluation of quadratic effects of the factor (a_w_ * a_w_, OA * OA and pH * pH) and interaction effects between factors (a_w_ * OA, a_w_ * pH and OA * pH).

**Table 5:** ANOVA results for the RSM and optimal conditions of TTD at OD_600nm_ (0.25, 0.5, 0.75, 1, 1.25, 1.5, 1.75, and 2) of *A. flavus*.

Factors	TTD							
	0.25	0.5	0.75	1	1.25	1.5	1.75	2
*p*-values ^a^								
a_w_	0.000	0.000	0.000	0.000	0.000	0.000	0.000	0.000
OA	0.737	0.288	0.574	0.302	0.226	0.062	0.109	0.104
pH	0.687	0.827	0.406	0.819	0.845	0.858	0.728	0.669
a_w_ * a_w_	0.000	0.000	0.000	0.000	0.000	0.000	0.002	0.029
a_w_ * OA	0.274	0.203	0.827	0.878	0.787	0.679	0.491	0.478
a_w_ * pH	0.532	0.790	0.471	0.847	0.534	0.560	0.294	0.309
OA * OA	0.007	0.026	0.026	0.112	0.162	0.091	0.182	0.314
OA * pH	0.226	0.473	0.186	0.079	0.057	0.086	0.045	0.094
pH * pH	0.914	0.475	0.761	0.354	0.275	0.096	0.175	0.331
R^2^	99.92	99.85	99.87	99.81	99.72	99.27	98.89	97.61
Adjusted R^2^	99.78	99.59	99.65	99.46	99.20	97.94	96.89	93.30
Optimisation (MIN) ^b^								
a_w_	0.985	0.985	0.985	0.986	0.986	0.990	0.990	0.990
OA (mM)	1.47	1.47	1.47	1.22	1.00	1.14	1.00	1.01
pH	5.60	5.60	5.92	5.75	5.60	5.78	5.60	5.60
Optimal value ^c^	2442	3088	3640	4055	4351	4569	5218	6261
Optimisation (MAX) ^b^								
a_w_	0.920	0.920	0.920	0.920	0.920	0.920	0.920	0.920
OA (mM)	2.00	2.00	2.00	2.00	2.00	2.00	2.00	2.00
pH	5.60	5.75	5.60	5.60	5.60	5.60	5.60	5.61
Optimal value ^c^	9985	12,828	14,201	14,746	15,149	15,841	16,068	16,123

^a^ *p*-values lower than 0.05 are statistically significant. Green values indicate positive effects, and red values indicate negative effects. ^b^ Optimisation means the combination of significant factors necessary to obtain the highest value (max) or the lowest value of TTD (min). ^c^ minutes. * Evaluation of quadratic effects of the factor (a_w_ * a_w_, OA * OA and pH * pH) and interaction effects between factors (a_w_ * OA, a_w_ * pH and OA * pH).

**Table 6:** ANOVA results for the RSM and optimal conditions of OTA and AFs production by *A. welwitschiae* and *A. flavus*, respectively.

Factors	OTA	AFB_1_	AFB_2_
*p*-values ^a^			
a_w_	0.006	0.005	0.014
OA	0.890	0.402	0.362
pH	0.320	0.209	0.652
a_w_ * a_w_	0.128	0.637	0.043
a_w_ * OA	0.850	0.684	0.797
a_w_ * pH	0.117	0.112	0.475
OA * OA	0.614	0.922	0.791
OA * pH	0.700	0.759	0.892
pH * pH	0.177	0.635	0.596
R^2^	86.43	85.45	82.67
Adjusted R^2^	62.00	59.27	51.46
Lack-of-fit test (*p*-value)	0.116	0.141	0.343
Optimisation (MIN) ^b^			
a_w_	0.920	0.920	0.920
OA (mM)	2.00	2.00	1.83
pH	5.88	5.60	5.84
Optimal value ^c^	0	0	31
Optimisation (MAX) ^b^			
a_w_	0.990	0.990	0.978
OA (mM)	1.73	1.00	1.00
pH	5.60	5.60	5.60
Optimal value ^c^	6556	25558	604

^a^ *p*-values lower than 0.05 are statistically significant. Green values indicate positive effects, and red values indicate negative effects. ^b^ Optimisation means the combination of significant factors necessary to obtain the highest value (max) or the lowest value of mycotoxin (min). ^c^ µg/kg. * Evaluation of quadratic effects of the factor (a_w_ * a_w_, OA * OA and pH * pH) and interaction effects between factors (a_w_ * OA, a_w_ * pH and OA * pH).

**Table 7:** Prediction equations taking into account the significant independent variables to determine growth parameters (TTD, lag phase, and µ_max_) and mycotoxin (OTA, AFB_1_, and AFB_2_) production by *A. welwitschiae* and *A. flavus* using RSM.

Mould Species	Growth Parameter/Mycotoxin	Prediction Equation	Correlation Coefficients (R^2^)
*A. welwitschiae*	TTD OD_600nm_ 0.25	+702,950 − 1.41273 × 10^6^ * a_w_ − 428.95 * OA + 712,686 * a_w_^2^ + 5.82077 × 10^−10^ * a_w_ * OA + 7.567 × 10^−10^ * a_w_ * pH + 1.16415 × 10^−10^ * OA^2^ + 5.23869 × 10^−10^ * OA * pH − 1.16415 × 10^−10^ * pH^2^	97.89%
	TTD OD_600nm_ 0.5	+900,496 − 1.81156 × 10^6^ * a_w_ + 2.32831 × 10^−10^ * OA + 2.32831 × 10^−10^ * pH + 914,316 * a_w_^2^ − 1.28057 × 10^−9^ * a_w_ * OA − 1.28057 × 10^−9^ * a_w_ * pH + 1.16415 × 10^−10^ * OA^2^ − 8.14907 × 10^−10^ * OA * pH + 1.16415 × 10^−10^ * pH^2^	99.12%
	TTD OD_600nm_ 1	+1.02028 × 10^6^ − 2.08234 × 10^6^ * a_w_ + 8394.43 * OA + 2116.24 * pH + 1.0536 × 10^6^ * a_w_^2^ + 7.567 × 10^−10^ * a_w_ * OA + 8.73115 × 10^−10^ * a_w_ * pH − 5.23869 × 10^−10^ * OA^2^ − 1410.83 * OA * pH − 3.49246 × 10^−10^ * pH^2^	99.45%
	Lag phase (hours)	+12,448.4 – 24,732.0 * a_w_ + 12,325.1 * a_w_^2^	99.43%
	µ_max_ (OD_600nm/day_)	−820.781 + 1802.53 * a_w_ − 24.225 * OA − 6.10714 * pH − 943.732 * a_w_^2^ + 4.07143 * OA * pH	89.45%
	OTA	−53,690.8 + 57,856.6 * a_w_	56.52%
*A. flavus*	TTD OD_600nm_ 0.25	+1.60294 × 10^6^ − 3.24384 × 10^6^ * a_w_ − 3623.33 * OA + 2.32831 × 10^−10^ * pH + 1.64645 × 10^6^ * a_w_^2^ − 1.5134 × 10^−9^ * a_w_ * OA − 1.16415 × 10^−10^ * a_w_ * pH + 1207.78 * OA^2^ − 1.16415 × 10^−9^ * OA * pH − 3.49246 × 10^−10^ * pH^2^	99.85%
	TTD OD_600nm_ 1	+2.1616 × 10^6^ − 4.37294 × 10^6^ * a_w_ + 5.82077 × 10^−10^ * OA + 5.82077 × 10^−10^ * pH + 2.21607 × 10^6^ * a_w_^2^ − 1.62981 × 10^−9^ * a_w_ * OA − 1.62981 × 10^−9^ * a_w_ * pH + 2.32831 × 10^−10^ * OA^2^ − 1.16415 × 10^−9^ * OA * pH + 2.32831 × 10^−10^ * pH^2^	99.39%
	TTD OD_600nm_ 1.75	+1.36815 × 10^6^ − 2.81425 × 10^6^ * a_w_ + 25,603.5 * OA + 6454.67 * pH + 1.41406 × 10^6^ * a_w_^2^ − 8.14907 × 10^−10^ * a_w_ * OA − 8.14907 × 10^−10^ * a_w_ * pH − 5.82077 × 10^−10^ * OA^2^ − 4303.11 * OA * pH + 2.32831 × 10^−10^ * pH^2^	96.58%
	Lag phase (hours)	+22,787.2 – 46,263.2 * a_w_ + 429.75 * OA + 23,529.2 * a_w_^2^ − 450.0 * a_w_ * OA	99.09%
	µ_max_ (OD_600nm/day_)	−273.546 + 566.037 * a_w_ − 291.399 * a_w_^2^	90.32%
	AFB_1_	−185,568 + 202,733 * a_w_ + 1.45519 × 10^−10^ * a_w_ * OA + 1.45519 × 10^−10^ * a_w_ * pH + 2.03727 × 10^−10^ * OA * pH	63.99%
	AFB_2_	−145,661 + 300,983 * a_w_ – 154,972 * a_w_^2^ − 1.74623 × 10^−10^ * a_w_ * OA − 1.74623 × 10^−10^ * a_w_ * pH − 1.16415 × 10^−10^ * OA * pH	74.68%

## Data Availability

The original contributions presented in this study are included in the article/Supplementary Materials. Further inquiries can be directed to the corresponding author.

## Supplementary Materials

The following supporting information can be downloaded at: https://www.mdpi.com/article/10.3390/foods14223854/s1. Table S1: Means and deviations of values of lag phase (hours), µ_max_ (OD_600nm/day_), and mycotoxin (µg/kg) of *A. welwitschiae* and *A. flavus*, respectively, for 15 tested conditions. Table S2: Data on the means and deviations of TTD (min) at different values of OD_600nm_ of *A. welwitschiae* for 15 tested conditions. Table S3: Data on the means and deviations of TTD (min) at different values of OD_600nm_ of *A. flavus* for 15 tested conditions. Table S4: Means and deviations of experimental and predicted values of lag phase (hours) and growth rate (µ_max_; OD_600nm/day_) of *A. welwitschiae* and *A. flavus*. Table S5: Data on the means and deviations of experimental and predicted values of TTD at different values of OD_600nm_ of *A. welwitschiae* and *A. flavus*. Table S6: Means and deviations of experimental and predicted values of OTA and aflatoxins of *A. welwitschiae* and *A. flavus*. Figure S1: *A. flavus* and *A. welwitschiae* growing on Bioscreen C plates under 15 conditions.

## Abbreviations

The following abbreviations are used in this manuscript:

a_w_	Water activity
OA	Oxalic acid
RSM	Response Surface Methodology
HPLC-FLD	High-performance liquid chromatography—fluorescence detector
OTA	Ochratoxin A
AFs	Aflatoxins
AFB_1_	Aflatoxin B_1_
AFB_2_	Aflatoxin B_2_
R^2^	R-squared
TTD	Time to detection
OD	Optical density
µ_max_	Growth rate
BBD	Box–Behnken design
SSFB	Semi-solid fig-based
ANOVA	Analysis of variance
RSQM	Response Surface Quadratic Model
MAX	Maximise
MIN	Minimise
<LOD	Below of limit of detection
