# Supplementary material for: Modelling the Combined Effects of Oxalic Acid, Water Activity, and pH on the Growth and Mycotoxin Production of Aspergillus spp. in a Dried Fig System
Source: Foods. 2025 Nov 11;14(22):3854. doi: 10.3390/foods14223854 (PMC12651421; doi:10.3390/foods14223854)
Supplement: Supplementary file 1 [file foods-14-03854-s001.zip › foods-3933216-supplementary.pdf]

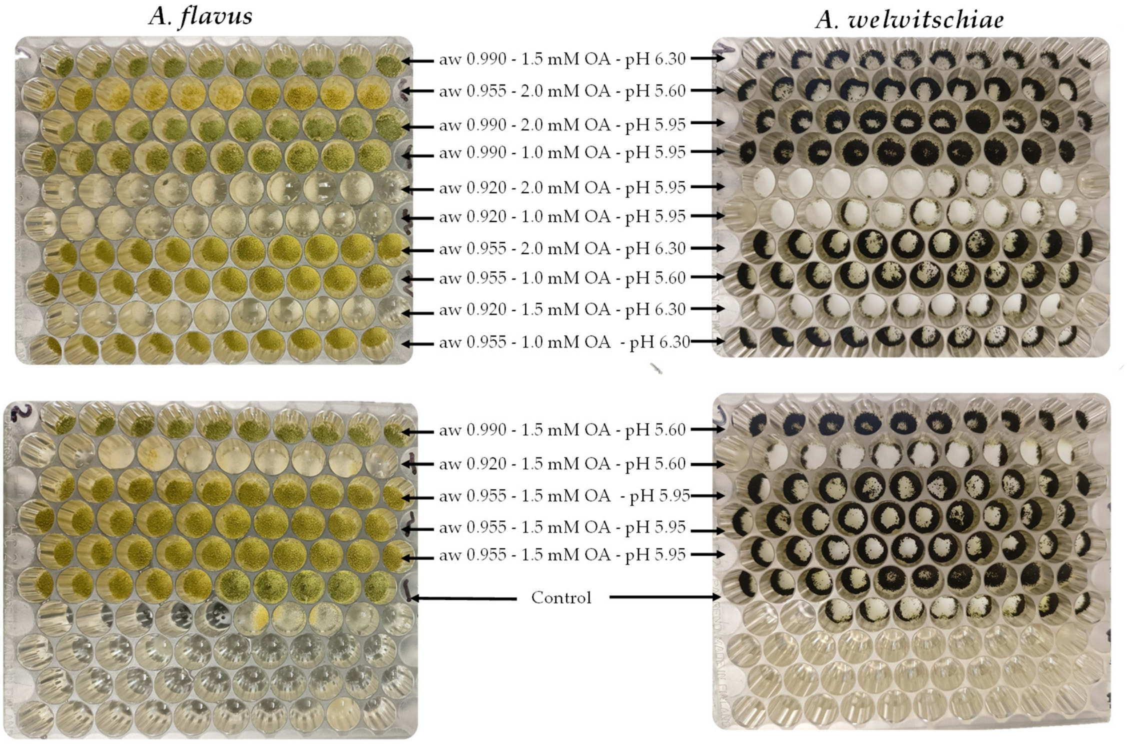

Figure S1. *A. flavus* and *A. welwitschiae* growing on Bioscreen C plates under 15 conditions.

Table S1. Means and deviations of values of lag phase (hours), µmax ($OD_{600\,nm}$/day), and mycotoxin (µg/kg) of *A. welwitschiae* and *A. flavus*, respectively, for 15 tested conditions.

			A. welwitschiae									***A. flavus***												
**a_w**	**AO (mM)**	**pH**	**lag (hours)**			**µmax (OD_{600nm}/day)**			**OTA (µg/kg)**		**lag (hours)**			**µmax (OD_{600nm}/day)**			**AFB_1 (µg/kg)**			**AFB_2 (µg/kg)**		
			Mean		SD	Mean		SD	Mean	SD	Mean		SD	Mean		SD	Mean		SD	Mean		SD
0.990	1.5	6.30	45.1	±	3.6	2.36	±	0.30	3145.0	1261.6	23.6	±	2.4	1.12	±	0.06	6394.8	±	2195.1	257.2	±	74.9
0.955	2.0	5.60	72.7	±	3.0	2.71	±	0.30	1054.4	578.5	69.9	±	6.9	1.07	±	0.13	4366.9	±	1776.9	330.4	±	154.7
0.990	2.0	5.95	47.2	±	2.9	2.42	±	0.19	2989.3	616.5	20.2	±	7.0	1.09	±	0.08	13774.7	±	6979.4	415.3	±	176.4
0.990	1.0	5.95	42.5	±	1.7	2.42	±	0.06	2492.7	355.0	21.2	±	3.3	1.24	±	0.08	17035.1	±	1541.4	462.8	±	32.2
0.920	2.0	5.95	126.6	±	1.9	2.42	±	0.16	73.3	22.2	179.1	±	5.1	0.53	±	0.06	956.0	±	391.1	51.5	±	15.9
0.920	1.0	5.95	126.2	±	1.8	2.65	±	0.13	75.6	9.0	148.6	±	0.0	0.58	±	0.00	519.1	±	286.5	27.0	±	12.2
0.955	2.0	6.30	71.8	±	1.4	4.58	±	0.28	921.9	384.1	64.3	±	8.6	1.30	±	0.18	7665.6	±	3454.7	420.7	±	239.9
0.955	1.0	5.60	68.7	±	1.0	3.74	±	0.44	1049.5	270.8	65.0	±	4.5	1.35	±	0.13	9879.6	±	4291.6	487.9	±	138.2
0.920	1.5	6.30	131.1	±	1.4	2.59	±	0.32	84.4	12.3	165.7	±	10.6	0.58	±	0.00	909.8	±	585.3	61.4	±	34.4
0.955	1.0	6.30	67.6	±	1.2	2.76	±	0.19	1940.2	740.3	70.1	±	6.7	1.30	±	0.14	10401.4	±	2068.7	616.2	±	102.5
0.990	1.5	5.60	39.3	±	1.5	2.04	±	0.12	7856.6	1711.0	30.7	±	4.3	1.47	±	0.12	25253.4	±	3746.4	558.8	±	71.8
0.920	1.5	5.60	123.7	±	1.6	2.56	±	0.28	50.6	3.3	161.6	±	8.6	0.58	±	0.10	3307.7	±	822.1	158.1	±	29.8
0.955	1.5	5.95	68.8	±	1.0	3.60	±	0.51	584.7	62.8	61.6	±	3.6	1.21	±	0.13	4778.6	±	1277.9	303.7	±	83.9
0.955	1.5	5.95	71.2	±	0.5	3.84	±	0.60	370.2	98.3	59.1	±	3.4	1.24	±	0.08	6439.0	±	2317.6	402.4	±	141.5
0.955	1.5	5.95	70.0	±	2.1	3.89	±	0.52	745.5	202.2	65.1	±	4.0	1.32	±	0.06	8949.9	±	1379.8	510.6	±	96.1

Table S2. Data on the means and deviations of TTD (min) at different values of $OD_{600\ nm}$ of *A. welwitschiae* for 15 tested conditions.

a_w	AO (mM)	pH	TTD OD 0.25		TTD OD 0.50		TTD OD 0.75		TTD OD 1.00		TTD OD 1.25		TTD OD 1.50		TTD OD 1.75		TTD OD 2.00	
			Mean	SD	Mean	SD	Mean	SD	Mean	SD	Mean	SD	Mean	SD	Mean	SD	Mean	SD
0.990	1.5	6.30	2219.0[a]	± 150.4	3144.1	± 114.9	3647.0	± 69.3	4026.6	± 66.8	4485.8	± 72.2	4821.5	± 39.1	5136.4	± 18.5	5492.6	± 41.7
0.955	2.0	5.60	2926.9	± 138.8	4446.0	± 220.1	5263.2	± 151.1	5645.6	± 315.8	5873.6	± 363.8	6162.0	± 333.9	6568.6	± 246.8	7177.2	± 254.8
0.990	2.0	5.95	2305.0	± 65.6	3391.6	± 37.5	3817.7	± 31.2	4111.1	± 75.9	4337.5	± 126.4	4691.2	± 156.2	5092.9	± 121.3	5688.5	± 135.8
0.990	1.0	5.95	2318.0	± 125.8	3224.9	± 125.7	3686.8	± 77.1	3935.2	± 83.7	4089.4	± 102.5	4312.4	± 146.4	4744.4	± 136.1	5217.6	± 108.5
0.920	2.0	5.95	5337.8	± 228.9	7567.4	± 197.1	8520.5	± 76.1	8882.8	± 106.5	9205.9	± 78.1	9473.9	± 68.6	9850.3	± 83.1	11351.7	± 548.4
0.920	1.0	5.95	6092.5	± 270.0	7877.1	± 158.9	8535.7	± 86.5	8867.4	± 126.4	9156.9	± 103.8	9399.0	± 76.2	9747.1	± 90.9	11384.8	± 1236.0
0.955	2.0	6.30	3046.3	± 234.2	4425.1	± 127.2	4855.8	± 56.8	4982.6	± 49.7	5082.1	± 47.8	5206.8	± 50.2	5444.5	± 62.2	5971.0	± 144.0
0.955	1.0	5.60	3441.1	± 119.8	4330.4	± 94.3	4882.2	± 88.6	5033.1	± 107.1	5163.5	± 125.5	5310.7	± 144.3	5583.3	± 186.1	6139.7	± 259.9
0.920	1.5	6.30	6290.7	± 267.2	8061.8	± 132.2	8871.7	± 207.2	9173.5	± 183.8	9404.4	± 174.8	9639.7	± 203.2	10101.3	± 275.7	12481.3	± 1264.7
0.955	1.0	6.30	3480.1	± 119.0	4269.0	± 85.0	5009.7	± 53.7	5357.7	± 62.2	5545.5	± 76.0	5787.2	± 113.5	6234.3	± 100.2	6823.1	± 145.0
0.990	1.5	5.60	1987.1	± 96.4	2914.2	± 76.4	3535.0	± 53.2	3913.7	± 47.3	4161.8	± 52.0	4511.4	± 99.6	5009.5	± 77.3	5559.1	± 115.1
0.920	1.5	5.60	5528.0	± 230.1	7430.1	± 124.2	8348.2	± 104.8	8650.2	± 160.1	8975.5	± 199.2	9269.2	± 183.2	9631.2	± 142.7	10979.1	± 173.3
0.955	1.5	5.95	2963.7	± 117.9	4190.3	± 75.2	4832.6	± 101.1	5012.5	± 160.0	5155.6	± 188.4	5319.3	± 219.2	5588.9	± 264.7	6147.8	± 289.0
0.955	1.5	5.95	3103.2	± 217.3	4331.6	± 123.8	4852.7	± 27.6	4994.1	± 26.7	5126.3	± 28.5	5290.6	± 35.4	5570.5	± 55.7	6279.4	± 47.9
0.955	1.5	5.95	2995.9	± 240.7	4327.2	± 114.3	4902.4	± 76.0	5055.2	± 87.6	5195.4	± 98.0	5357.2	± 102.4	5662.0	± 115.4	6560.3	± 186.4

[a]min.

Table S3. Data on the means and deviations of TTD (min) at different values of $OD_{600\ nm}$ of *A. flavus* for 15 tested conditions.

a_w	AO (mM)	pH	TTD OD 0.25		TTD OD 0.50		TTD OD 0.75		TTD OD 1.00		TTD OD 1.25		TTD OD 1.50		TTD OD 1.75		TTD OD 2.00	
			Mean	SD	Mean	SD	Mean	SD	Mean	SD	Mean	SD	Mean	SD	Mean	SD	Mean	SD
0.990	1.5	6.30	2650.6[a]	± 83.2	3480.8	± 114.2	3962.7	± 110.5	4471.4	± 199.9	5075.8	± 118.8	5671.0	± 144.4	6968.0	± 108.0	8298.4	± 73.2
0.955	2.0	5.60	4453.9	± 167.4	5608.4	± 175.3	6558.5	± 193.0	7457.2	± 321.1	8051.7	± 274.1	9660.3	± 1932.9	10798.6	± 684.5	12306.2	± 889.0
0.990	2.0	5.95	2627.2	± 124.3	3441.3	± 246.2	3937.5	± 193.0	4479.4	± 270.9	5051.3	± 191.6	5652.1	± 298.0	6853.3	± 366.3	8238.8	± 346.5
0.990	1.0	5.95	2820.5	± 70.3	3570.1	± 71.7	4007.8	± 54.3	4382.8	± 89.4	4853.9	± 66.5	5191.7	± 60.4	6008.8	± 141.2	7161.9	± 186.0
0.920	2.0	5.95	9901.9	± 262.1	12972.0	± 272.3	14204.3	± 48.4	14224.7	± 1.6	14228.3	± 2.0	14231.9	± 2.5	14235.6	± 3.1	14239.2	± 3.6
0.920	1.0	5.95	9769.3	± 349.1	12422.8	± 233.5	14163.8	± 60.4	14223.0	± 0.7	14226.0	± 0.7	14229.0	± 0.8	14232.1	± 0.9	14235.1	± 0.9
0.955	2.0	6.30	4154.4	± 100.9	5336.5	± 116.4	6093.5	± 143.8	6443.9	± 129.1	6691.6	± 150.3	7993.2	± 2582.3	8337.0	± 228.4	9642.1	± 230.7
0.955	1.0	5.60	4173.4	± 110.5	5250.0	± 101.9	5970.0	± 159.3	6382.6	± 148.0	6642.6	± 161.2	7021.4	± 293.9	8158.3	± 363.3	9434.0	± 326.9
0.920	1.5	6.30	9326.4	± 353.2	11946.5	± 396.4	13898.2	± 294.5	14374.7	± 66.9	14375.1	± 65.9	14375.5	± 64.9	14375.8	± 63.9	14376.2	± 63.0
0.955	1.0	6.30	4240.6	± 109.5	5337.4	± 94.9	6242.8	± 96.6	6662.0	± 120.3	6964.9	± 159.4	7570.4	± 286.3	8708.8	± 311.0	9660.1	± 291.2
0.990	1.5	5.60	2525.5	± 78.9	3248.1	± 81.0	3745.0	± 66.3	4064.0	± 68.1	4428.1	± 136.0	4926.5	± 74.5	5642.0	± 155.5	6735.7	± 125.8
0.920	1.5	5.60	9379.6	± 572.6	11844.1	± 516.4	13305.7	± 505.0	13847.7	± 329.7	14184.5	± 124.7	14279.6	± 61.1	14379.5	± 52.3	14400.0	± 0.0
0.955	1.5	5.95	4009.0	± 75.7	5141.5	± 110.3	5843.6	± 219.1	6308.5	± 245.1	6613.9	± 297.0	7002.4	± 483.5	8157.0	± 649.1	9589.7	± 537.2
0.955	1.5	5.95	3888.5	± 57.8	4996.1	± 56.2	5646.4	± 54.7	6211.4	± 116.3	6530.3	± 168.4	6902.4	± 382.7	8026.1	± 511.4	9370.5	± 357.4

0.955	1.5	5.95	3988.7	±	57.0	5154.2	±	74.8	5866.2	±	125.6	6340.9	±	87.2	6588.3	±	100.9	6919.7	±	186.1	8047.0	±	277.4	9420.1	±	351.8

[a]min.

Table S4. Means and deviations of experimental and predicted values of lag phase (hours) and growth rate (μmax; $OD_{600\ nm}$/day) of *A. welwitschiae* and *A. flavus*.

Validation points			*A. welwitschiae*								*A. flavus*							
			Lag phase (hours)				μmax ($OD_{600\ nm}$/day)				Lag phase (hours)				μmax ($OD_{600\ nm}$/day)			
			Experimental data			Predicted data	Experimental data			Predicted data	Experimental data			Predicted data	Experimental data			Predicted data
pH	a_w	OA (mM)	Mean		SD	Mean	Mean		SD	Mean	Mean		SD	Mean	Mean		SD	Mean
6.00	0.94	1.25	89.2	±	1.9	90.8	4.3	±	0.3	3.3	81.7	±	2.7	98.6	1.3	±	0.1	1.0
6.00	0.97	1.25	45.6	±	7.3	55.0	3.0	±	0.6	3.3	35.0	±	2.0	42.1	1.5	±	0.1	1.3
6.00	0.94	1.75	92.5	±	2.6	90.8	4.1	±	0.4	3.4	79.1	±	3.2	102.0	1.0	±	0.1	1.0
6.00	0.97	1.75	49.8	±	2.9	55.0	3.3	±	0.3	3.4	39.9	±	5.1	38.7	1.8	±	0.1	1.3
6.15	0.95	1.50	77.6	±	1.7	76.4	4.8	±	0.6	3.6	64.3	±	9.8	75.6	1.5	±	0.3	1.2
R values			0.978				0.524				0.960				0.738			

Table S5. Data on the means and deviations of experimental and predicted values of TTD at different values of $OD_{600\ nm}$ of *A. welwitschiae* and *A. flavus*.

Validation points			*A. welwitschiae*											
			TTD $OD_{600\ nm}$ 0.25				TTD $OD_{600\ nm}$ 0.50				TTD $OD_{600\ nm}$ 1.00			
			Experimental data			Predicted data	Experimental data			Predicted data	Experimental data			Predicted data
pH	a_w	OA (mM)	Mean		SD	Mean	Mean		SD	Mean	Mean		SD	Mean
6.00	0.94	1.25	4810.3[a]	±	138.4	4177.0	5642.9	±	105.8	5519.2	6170.6	±	100.4	6450.6
6.00	0.97	1.25	3121.3	±	382.4	2632.0	3532.9	±	512.2	3562.7	3869.0	±	536.8	4351.7
6.00	0.94	1.75	4818.5	±	152.2	3962.5	5810.9	±	233.6	5519.2	6431.0	±	73.8	6415.3
6.00	0.97	1.75	3024.6	±	214.8	2417.5	3590.7	±	158.4	3562.7	4129.6	±	207.2	4316.4
6.15	0.95	1.50	4220.6	±	152.1	3412.2	5000.2	±	108.1	4684.2	5366.0	±	50.4	5522.6
R values			0.959				0.966				0.967			

Validation points			*A. flavus*											
			TTD $OD_{600\ nm}$ .25				TTD $OD_{600\ nm}$ 1.00				TTD $OD_{600\ nm}$ 1.75			
			Experimental data			Predicted data	Experimental data			Predicted data	Experimental data			Predicted data
pH	a_w	OA (mM)	Mean		SD	Mean	Mean		SD	Mean	Mean		SD	Mean
6.00	0.94	1.25	5136.4	±	94.6	5891.6	7308.6	±	221.7	9155.9	9341.2	±	353.5	10677.5
6.00	0.97	1.25	3098.1	±	96.3	2918.0	4552.8	±	132.9	4948.5	5445.3	±	70.9	7275.6
6.00	0.94	1.75	4905.4	±	107.1	5891.6	8058.3	±	285.0	9155.9	11408.1	±	1313.2	10569.9
6.00	0.97	1.75	3040.5	±	127.3	2918.0	4288.2	±	100.8	4948.5	5249.8	±	93.8	7168.0
6.15	0.95	1.50	4108.1	±	74.3	4495.6	6418.0	±	75.3	7310.2	8567.2	±	408.5	9206.9
R values			0.991				0.979				0.927			

[a]minutes

Table S6. Means and deviations of experimental and predicted values of OTA and aflatoxins of *A. welwitschiae* and *A. flavus*.

Validation points	*A. welwitschiae*	*A. flavus*	
	OTA (μg/kg)	AFB_1 (μg/kg)	AFB_2 (μg/kg)

			Experimental data			Predicted data	Experimental data			Predicted data	Experimental data			Predicted data
pH	aw	OA (mM)	Mean		SD	Mean	Mean		SD	Mean	Mean		SD	Mean
6.00	0.94	1.25	1517.2	±	282.5	694.4	12529.1	±	1439.0	5001.0	499.1	±	51.6	329.8
6.00	0.97	1.25	7616.7	±	629.1	2430.1	55678.0	±	10005.3	11083.0	1348.8	±	260.5	479.4
6.00	0.94	1.75	1167.9	±	172.3	694.4	25955.7	±	10767.7	5001.0	1060.5	±	438.6	329.8
6.00	0.97	1.75	5875.4	±	1552.8	2430.1	75792.3	±	18225.5	11083.0	1521.3	±	378.9	479.4
6.15	0.95	1.50	3331.6	±	124.5	1273.0	87939.0	±	17980.6	7028.4	4133.0	±	1462.7	410.6
R values					0.945				0.584				0.242	
